# CXCL12 Mediates Trophic Interactions between Endothelial and Tumor Cells in Glioblastoma

**DOI:** 10.1371/journal.pone.0033005

**Published:** 2012-03-12

**Authors:** Shyam Rao, Rajarshi Sengupta, Eun Joo Choe, B. Mark Woerner, Erin Jackson, Tao Sun, Jeffrey Leonard, David Piwnica-Worms, Joshua B. Rubin

**Affiliations:** 1 Department of Radiation Oncology, Washington University School of Medicine, St. Louis, Missouri, United States of America; 2 Department of Pediatrics, Washington University School of Medicine, St. Louis, Missouri, United States of America; 3 Bridging Research with Imaging, Genomics and High-Throughput Institute, Washington University School of Medicine, St. Louis, Missouri, United States of America; 4 Molecular Imaging Center, Mallinckrodt Institute of Radiology, Washington University School of Medicine, St. Louis, Missouri, United States of America; 5 Department of Neurosurgery, Washington University School of Medicine, St. Louis, Missouri, United States of America; 6 Department of Cell Biology & Physiology, Washington University School of Medicine, St. Louis, Missouri, United States of America; 7 Department of Developmental Biology, Washington University School of Medicine, St. Louis, Missouri, United States of America; 8 Department of Anatomy and Neurobiology, Washington University School of Medicine, St. Louis, Missouri, United States of America; Sanford-Burnham Medical Research Institute, United States of America

## Abstract

Emerging evidence suggests endothelial cells (EC) play a critical role in promoting Glioblastoma multiforme (GBM) cell proliferation and resistance to therapy. The molecular basis for GBM-EC interactions is incompletely understood. We hypothesized that the chemokine CXCL12 and its receptor CXCR4 could mediate direct interactions between GBM cells and tumor-associated endothelial cells and that disruption of this interaction might be the molecular basis for the anti-tumor effects of CXCR4 antagonists. We investigated this possibility *in vivo* and in an *in vitro* co-culture model that incorporated extracellular matrix, primary human brain microvascular ECs (HBMECs) and either an established GBM cell line or primary GBM specimens. Depletion of CXCR4 in U87 GBM cells blocked their growth as intracranial xenografts indicating that tumor cell CXCR4 is required for tumor growth *in vivo*. *In vitro*, co-culture of either U87 cells or primary GBM cells with HBMECs resulted in their co-localization and enhanced GBM cell growth. Genetic manipulation of CXCL12 expression and pharmacological inhibition of its receptors CXCR4 and CXCR7 revealed that the localizing and trophic effects of endothelial cells on GBM cells were dependent upon CXCL12 and CXCR4. These findings indicate that the CXCL12/CXCR4 pathway directly mediates endothelial cell trophic function in GBMs and that inhibition of CXCL12-CXCR4 signaling may uniquely target this activity. Therapeutic disruption of endothelial cell trophic functions could complement the structural disruption of anti-angiogenic regimens and, in combination, might also improve the efficacy of radiation and chemotherapy in treating GBMs.

## Introduction

Tissue architecture, including cell-cell and cell-extracellular matrix interactions, is an essential determinant of cellular behavior in normal and pathological states. The potential of three-dimensional tissue relationships to influence the phenotype of cancer cells has been appreciated since the time of Virchow and directly demonstrated more recently (reviewed in [1]). Increasingly, malignant behavior is being redefined as a consequence of not only mutational events in cancer cells but also specific tumor-stroma interactions that promote tumor formation, progression, and resistance to therapy [2].

The earliest histopathological descriptions of glioblastoma multiforme (GBM) recognized the involvement of vascular endothelial cells (ECs) in tumor architecture. The close apposition of glioblastoma cells to the abluminal surface of endothelial cells, a cardinal feature of GBM, was originally described by Scherer, who suggested this was a route by which tumor cells invaded surrounding brain [3].

In GBMs, there is an abundance of vascular endothelial cells, which was initially assumed to maintain the high metabolic rates observed in these tumors through the formation of perfusing blood vessels [4]. However, it is now clear that the vasculature within GBM is abnormal. Despite a redundancy in microvessels, tumors exist in a relatively hypoxic state in which they utilize anaerobic metabolism to a greater degree than normal brain tissue [5]. These observations call into question the role of the abundant vasculature in GBM.

Scherer suggested that the perivascular space possessed specialized properties important for the maintenance and spread of glioblastoma [3]. The details of this specialization have recently been suggested to include maintenance of a stem cell-like phenotype in glioblastoma cells localized to this region [6]. The stem-like phenotype includes increased DNA repair capacity, increased efflux pump expression and growth in response to endothelial cell-derived factor(s) [7], [8], [9], [10]. Thus, the peri-vascular space is predicted to contribute to tumor growth and therapeutic resistance. It is therefore imperative to understand the biology of this space and to develop systems in which to screen for drugs that can disrupt its function(s).

The vasculature of GBM is distinguished from normal brain vasculature by levels of CXCL12 expression. In extensive studies of malignant neural and astrocytic tumors, we and others found increased CXCL12 expression in the endothelium of tumor-associated blood vessels. [11], [12], [13], [14], [15], [16], [17]. The functional significance of increased endothelial expression of CXCL12 in GBMs remains to be fully defined. CXCL12 plays multiple roles during normal development and tissue homeostasis both inside and outside the central nervous system. Prominent among these roles is the regulation of progenitor cell localization to germinal niches [18], [19], [20], [21], [22], [23], [24], the regulation of progenitor cell proliferation [22], [25], [26], survival [27] and differentiation [28], as well as the regulation of immune cell chemotaxis and activation [29]. Relevant to brain tumors, endothelial cell CXCL12 could directly regulate tumor growth and spread, promote angiogenesis and/or regulate immune response to tumors.

CXCR4, a CXCL12 receptor, is expressed on brain tumor cells and high levels of expression have negative prognostic significance [30]. These findings suggest that the CXCL12/CXCR4 pathway functions in tumor growth and/or therapeutic resistance. The anti-tumor effects of AMD3100 and AMD3465, competitive antagonists of CXCR4 activation also indicate that CXCL12 regulation of tumor growth involves activation of tumor cell CXCR4 [14], [31], [32]. In intracranial xenograft models of GBM and medulloblastoma we demonstrated that systemic delivery of AMD3100 or AMD3465 had significant anti-tumor activity against established xenografts. Within 48 hours of the initiation of AMD3100 treatment, tumor cells exhibited decreased proliferation and increased apoptosis indicating that AMD3100 disrupted a critical direct effect of CXCL12 on tumor growth [14].

Recently, using a similar U87 intracranial xenograft model of GBM, AMD3100 was shown to prevent tumor recurrence after radiation therapy through inhibition of mesenchymal progenitor cell recruitment for vasculogenesis [33]. Together, these studies underscore the potential complexity of CXCL12 effects on brain tumor growth and the possibility for multiple mechanisms of CXCR4 antagonist action. In addition to CXCR4, CXCL12 also binds to a second G-protein coupled receptor CXCR7, which has been recently characterized. CXCR7 is shown to be expressed and functional in a number of glioma cell lines, although its exact role in tumorigenesis is still unclear [34], [35].

In order to better define the roles of CXCL12 and its receptors in functions of the GBM perivascular niche we investigated the interactions between human brain microvascular endothelial cells and GBM cells when the two cell types were cultured together. In this system, where there is no vasculogenesis and uniform oxygenation, we demonstrate that CXCL12 is essential for chemo-attracting GBM cells to the peri-endothelial cell space and mediating a direct trophic effect of endothelial cells on GBM cells. Endothelial cell effects on GBM cell growth were blocked by both the specific CXCR4 antagonist AMD3100 and knock-down of endothelial cell CXCL12 expression with short hairpin interfering RNA (shRNA). In contrast, the CXCR7 antagonists CCX733 and CCX773 had no effect on CXCL12-induced growth. These data indicate that targeting the CXCL12/CXCR4 pathway could abrogate a specialized trophic function of GBM-associated vasculature that contributes to brain tumor growth. These data further suggest that therapeutic regimens that combine antagonism of both perivascular niche formation and function could have synergistic effects on tumor growth and recurrence.

## Materials and Methods

### Chemicals, Reagents, and Antibodies

All chemicals were obtained from Sigma-Aldrich (St. Louis, MO) unless otherwise indicated. All tissue culture reagents and media were obtained from Invitrogen (Carlsbad, CA) unless otherwise indicated. A construct containing mCherry cDNA was the kind gift of Dr. Roger Y. Tsien (University of California, San Diego, CA). Antibodies utilized in this study were: PECAM/CD31 (Santa Cruz Biotechnology, Santa Cruz, CA), GFAP (Sigma-Aldrich), CXCR4 monoclonal (R&D Systems, Minneapolis, MN), CXCR4 polyclonal (Leinco, St. Louis, MO), CXCL12 (Peprotech, Rock Hill, NJ), β-actin (Sigma-Aldrich) and immunoglobulin (IgG) isotype controls (Jackson Immuno-Research, West Grove, PA). AMD3100 was purchased from Sigma-Aldrich. CCX733 was obtained from Chemocentryx (Mountain View, CA).

### Cell Culture

#### Primary Human GBM cells

Primary tumor specimens from three adult patients and one pediatric patient with GBM were retrieved from the Children's Discovery Institute Pediatric Brain Tumor Bank at Washington University School of Medicine in accordance with an Institutional Review Board–approved protocol for human research. Freshly resected tumors specimens were kept in cold low calcium artificial cerebrospinal fluid (low Ca^++^ aCSF: 124 mM NaCl, 5 mM KCl, 3.2 mM MgCl_2_, 0.1 mM CaCl_2_, 26 mM NaHCO_3_, 10 mM D-glucose). Samples were minced and placed in dissociation media (25 U/mL collagenase, 150 ug/mL hyaluronidase, trypsin, 0.2 mg/mL kynurenic acid, 1% penicillin/streptomycin in low Ca^++^ aCSF). Single cells were pelleted and washed in aCSF three times, then resuspended in tumor sphere media (TSM), (Dulbecco's Modified Eagle Media Nutrient Mix F-12 (DMEM/F-12, Invitrogen) supplemented with Glutamax (Invitrogen), 20 ng/mL epithelial growth factor (EGF, Sigma-Aldrich), 20 ng/mL basic fibroblastic growth factor (bFGF, Chemicon (Billerica, MA)), 1% penicillin/streptomycin, and 1×B-27 Serum-Free Supplement (Invitrogen).

#### Primary Human Endothelial Cells

Primary human brain microvascular endothelial cells (HBMEC) were obtained from ScienCell, Carlsbad, CA). Primary human umbilical vein endothelial cells (HUVEC) were obtained from American Type Culture Collection (ATCC, Manassas, VA). For certain experiments endothelial cells were engineered to express mCherry fluorescent protein via lentiviral infection by viral particles that were produced by the Viral Vectors Core Facility of The Hope Center for Neurological Diseases at Washington University School of Medicine followed by sorting for expression with a MoFLO high speed cell sorter. Expression vectors used in viral production contained a transgene for mCherry fluorescent protein under a CMV promoter. ECs were used between passages 3–8 and maintained in endothelial cell growth media (EGM-2MV (Lonza, Basel, Switzerland)) on gelatin-coated dishes.

#### Established Human GBM cell line

U87 cells were originally obtained from ATTC and were engineered at low passage (<5) to express a fusion protein of firefly luciferase and enhanced green fluorescent protein (eGFP) driven by the human ubiquitin C promoter after transduction with a lentivirus (FUW-FLG) described previously [31], [32], [36]. U87 cells expressing firefly luciferase-eGFP (U87-Luc) were sorted to purity based on GFP expression, expanded and stored at −150 degrees Celsius. All experiments were performed with U87-Luc cells at less than passage 15 (approx 4 months), post their acquisition from ATCC. U87 cells were maintained in DMEM supplemented with 10% fetal bovine serum.

### GBM – endothelial cell co-cultures

#### Co-localization studies

Fifteen thousand HBMEC or HUVEC cells were plated on coverslips (35 cm^2^) coated with Matrigel extracellular matrix (BD Biosciences, San Jose, CA) as per manufacturer's instructions and then grown in EGM-2MV. After 24 hours, either 15,000 U87 cells or 30,000 primary GBM cells per 35 mm^2^ were added to the cultures and grown in Serum Free DMEM. Unless mentioned otherwise, co-localization measurements were performed 24 hours following addition of tumor cells.

#### Growth assays

Growth assays were conducted with Matrigel-coated, 96 well tissue culture plates containing 3000 HBMECs and 3000 U87 or 6000 primary GBM cells per well, plated and grown as described above.

### Primary Human GBM Tissue

Formalin-fixed, paraffin-embedded archival specimens of GBM were retrieved from the pathology files at Washington University School of Medicine in accordance with an Institutional Review Board–approved protocol for human research.

### Overexpression and knockdown

For knockdown experiments, CXCL12 or CXCR4 specific shRNAs (CXCL12: TGTGCATTGACCCGAAG CTAA or GAGTACCTGGAGAAAGCTTTA; CXCR4: GCTGCCTTACTACATTGGGAT) or a scrambled (control) shRNA sequence (CCGGCAACAAGATGAAGAGCACCAA) cloned into a lentiviral packaging vector (pLKO.1) were obtained from the Genome Institute at the Washington University School of Medicine in St Louis. Viral particles were produced from each packaging vector separately by the Viral Vectors Core Facility of The Hope Center for Neurological Diseases at Washington University School of Medicine. HBMECs were infected with lentivirus encoding either control or CXCL12 shRNA and U87 cells were infected with lentivirus encoding either control or CXCR4 shRNA as previously described [37]. Cells expressing shRNA constructs were selected using puromycin. As described in the results, knockdown of CXCL12 expression levels were determined by PCR and ELISA analysis. Knockdown of CXCR4 was confirmed by western blot. For over-expression experiments, HUVECs were infected with lentivirus containing CXCL12 gene as described previously [38]. CXCL12 overexpression was confirmed by ELISA.

### Quantitative PCR

RNA was isolated from endothelial cells using the RNeasy system (Qiagen (Valenica, CA)). Copy DNA was synthesized from 100 ng of RNA using iScript RTase, and CXCL12 and glyceraldehyde-3-phosphate dehydrogenase (GAPDH) transcripts were amplified using the power SYBR GREEN PCR Master Mix (Applied Biosystems (Carlsbad, CA)) according to the manufacturer's instructions. Primers for CXCL12 (forward primer, ATGCCCATGCCGATTCTT; reverse primer, GCCGGGCTACAATCTGAAGG) and GAPDH (forward primer, GGCAAATTCAACGGCACAGT; reverse primer, AGATGGTGATGGGCTTCCC) were obtained from Integrated DNA Technologies (Iowa City, IA) and used at 300 nmol/L. Samples were run in triplicate with a corresponding GAPDH control for each sample. PCR and data collection were done using the BioRad MiniOpticon Real Time PCR machine and Opticon Monitor 3 Software from BioRad (Hercules, CA). Relative transcript copy number for each CXCL12 and corresponding GAPDH sample were calculated using the delta-delta-C(t) method. The CXCL12 relative expression values for each condition were normalized to that of the lowest relative expression level for each experiment (n = 3).

### CXCL12 ELISA

HBMEC or HUVECs (wild type or infected with viruses as described in results) were plated onto gelatin or Matrigel coated dishes and maintained in EGM-2MV media for 24 hours. The media was then changed to Serum Free DMEM. After 48 hours of additional incubation, culture supernatants were collected and concentrated (100×) for CXCL12 detection by indirect ELISA assay. Briefly, CXCL12 standards, culture supernatants from endothelial cells and DMEM from Matrigel alone were incubated in a 96-well plate overnight in 20 mM sodium bicarbonate buffer (pH 9.5). The plate was blocked with 2% BSA for 1 hour and then incubated with rabbit anti-hCXCL12 (1∶200, PeproTech Inc.) for 1 hour. OPD substrate (Sigma-Aldrich) was added to the plate following incubation of goat anti-rabbit IgG HRP (1∶2500, Bio-Rad) for 1 hour. The reaction was stopped by 3M HCL and OD at 490 nm was measured with a microplate reader (Bio-Tek Instruments, Inc.). CXCL12 concentrations were calculated with reference to an ELISA standard curve. All samples were analyzed in duplicate.

### Western blot analysis

Protein extracts were obtained by lysing cells with lysis buffer [20 mmol/L Tris (pH 7.4), 137 mmol/L NaCl, 10% glycerol, and 1% Triton X-100] supplemented with Complete Protease Inhibitors (Roche) and Phosphatase Inhibitor Cocktail set #IV (Calbiochem (Gibbstown, NJ)). The proteins (25 µg) were resolved with 10% Bis-Tris gels (Invitrogen) and transferred onto the Hybond ECL nitrocellulose membrane (Amersham (Piscataway, NJ)) according to standard protocols. Blots were then probed with polyclonal anti-CXCR4 antibody. Total protein loading per lane was evaluated with anti-β-Actin antibody. This was followed by incubation with IRDye® conjugated secondary antibodies (LI-COR (Lincoln, NE)). Blots were imaged with the Odyssey fluorescent scanning system (Li-Cor).

### Immunofluorescence

Cells were fixed with 70% ethanol for 10 minutes at −20°C. Immunostaining of CXCL12, GFAP or CD31/PECAM was done as described [14]. CXCR7 antibody 11G8 was used (15 µg). Secondary AlexaFluor 488 or 568–conjugated donkey anti-mouse, AlexaFluor 555-conjugated donkey anti-rabbit, AlexaFluor 488–conjugated donkey anti-rat or anti-goat antibody were used at a concentration of 1∶1750 (Molecular Probes) for 90 minutes. Nuclei were counterstained with DAPI (4N, 6-diamidino-2-phenylindole) or TO-PRO 3 (Invitrogen).

### Tissue sections and immunohistochemistry

Sections (5 µm) were deparaffinized in xylene and rehydrated in descending alcohols to water. Endogenous peroxidase was blocked with 3% H_2_O_2_ in TBST [10 mmol/L Tris (pH 8.0), 0.15 mol/L NaCl, 0.05 Tween] and nonspecific avidin/biotin binding sites were blocked with the Vector Avidin/Biotin Blocking kit (Vector Laboratories, Burlingame, CA). Sections were additionally blocked with 10% serum from the animal source of the appropriate corresponding secondary antibody diluted in incubation media [0.1 mol/L Tris (pH 7.5), 0.15 mol/L NaCl, 2% nonfat dry milk, and 0.1% Triton X-100] and then incubated in primary antibody overnight at 4°C. CXCL12 was detected with rabbit polyclonal antibody (1∶66 dilution). Immunoreactive complexes were detected using the corresponding secondary biotin-conjugated antibodies augmented by streptavidin-horseradish peroxide and visualized by 3,3′-diaminobenzidine supplied by DAKO (Carpinteria, CA). Slides were then counterstained with hematoxylin, dehydrated through a series of alcohols and xylene, and coverslipped in 50∶50 xylene/Permount. Control sections were incubated with isotype-matched IgG.

### Localization Measurements

Primary human brain endothelial cells expressing mCherry and human GBM cells were grown on Matrigel coated coverslips, as above. After treatment, cells were fixed in 70% ethanol at −20°C for 10 minutes and counterstained with DAPI. Images were acquired on a Zeiss Scope.A1 fitted with an Axiocam MRc camera and using Axiovision software (Carl Zeiss, North America (Thornwood, NY)). Distances between GBM cell nuclei and the nearest endothelial cell nucleus were measured using a vector tool in Axiovision. For U87-HBMEC or HUVEC co-localization we measured ∼1000 cells in 4 separate experiments for each condition. We then used the average +/− SEM. For Primary GBM-HBMEC co-localization cells we measured ∼450 cells from 3 different patient specimens. We then used the average +/− SEM.

### Growth Assays

Primary GBM: After treatment, tumor cells were fixed and identified via immuno-labeling for glial fibrillary acidic protein (GFAP). Images were acquired using a Molecular Devices Image Express Micro High Content Imager at the High-Throughput Screening Core at Washington University in St. Louis. Automated image analysis using MetaExpress and AcuityExpress (Molecular Devices (Sunnyvale, CA)) was performed to count the total number of GFAP positive cells/well. Cell counts were normalized to control conditions in which GBM cells were cultured with Matrigel, but in the absence of ECs.

Established GBM cell line: For U87 cells expressing firefly luciferase, cell number was evaluated using bioluminescence measurements at the Molecular Imaging Core facility, BRIGHT Institute, Washington University in St. Louis. Endothelial cells did not express luciferase activity. Cell number, as determined by direct cell counts, and bioluminescence were linearly proportional under our culture conditions (data not shown). Cells were grown in 96 well black-wall clear-bottom plates (Costar). After treatment, cells were washed into phenol red-free media and exposed to 0.15 mg/ml luciferin for 10 minutes. Bioluminescence was measured using a charge-coupled device camera-based bioluminescence imaging system (IVIS 50; Caliper; exposure time 1–30 s, binning 8, field of view 12, f/stop 1, open filter). Regions-of-interest (ROIs) were drawn over images of wells and bioluminescence data, expressed as total photon flux (photons/s), were normalized to untreated controls [31], [36].

### TUNEL assays

Terminal nucleotidyl transferase–mediated nick end labeling (TUNEL) staining of fixed cells was done by standard procedures according to the manufacturer's directions (Roche Applied Science, Indianapolis, IN) along with nuclear counterstain. TUNEL-positive cells were detected under direct fluorescence microscopy. TUNEL positivity is reported as the percent of total GBM cell nuclei that were TUNEL positive.

### Generation of intracranial xenografts

Intracranial xenografts were generated as described previously. Homozygous NCR nude mice (Taconic Farms) were anesthetized [intraperitoneal ketamine (87 mg/kg)/xylazine (13 mg/kg); Phoenix Pharmaceuticals], the cranium was exposed, and a small hole was made 2 mm lateral and posterior to the bregma with a size 34 inverted cone burr (Dremel). Mice were positioned in a stereotactic frame (Stoelting) and 50,000 cells in 5 µL PBS were injected through a 27-gauge needle over 1 min at 3 mm below the dura mater. The incision was closed with Vetbond (3M).

### Bioluminescence imaging

NCR nude mice bearing intracranial xenografts of U87-Luc expressing scrambled shRNA (sc-U87-Luc) or CXCR4-specific shRNA (shCXCR4-U98-Luc) were injected with d-luciferin (150 µg/g; Biosynth) as previously described. After anesthesia using 2.5% isoflurane, mice were imaged with a charge-coupled device camera-based bioluminescence imaging system (IVIS 50; Xenogen; exposure time 1–30 s, binning 8, field of view 12, f/stop 1, open filter). Signals were displayed as photons/s/cm^2^/sr. Regions of interest were defined manually and images were processed using Living Image and IgorPro Software (Version 2.50) as described. Raw data were expressed as total photon flux (photons/s).

### Statistical Analyses

All values represent the means from experiments repeated at least 3 separate times. Data were analyzed using GraphPad Prism version 4.00 (GraphPad Software) using the specific statistical tests identified in the corresponding figure legends. Potential statistical outliers were detected by application of Grubb's test. A single animal was removed from the *in vivo* studies as an outlier. A second animal exhibited highly erratic bioluminescence and was also excluded. This did not alter the results.

## Results

**Figure 1:**
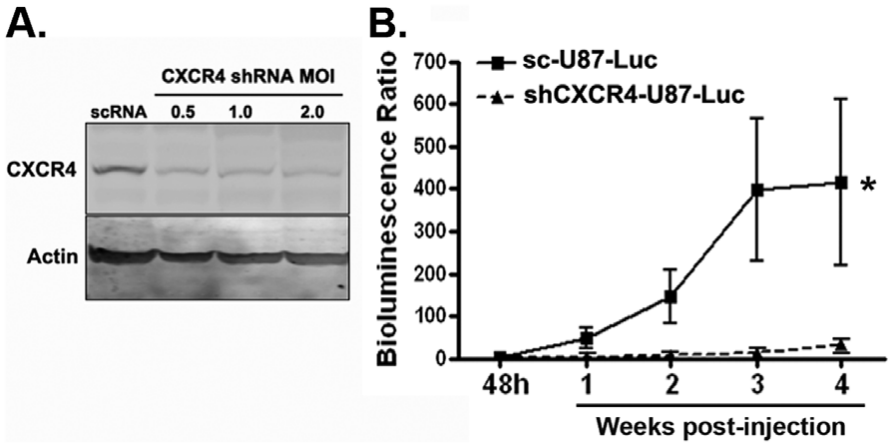
Deletion of CXCR4 suppresses the growth of intracranial U87 xenografts. (**A**) Western blot analysis of CXCR4 expression in U87 cells infected with lentirvirus encoding a scrambled shRNA control (scRNA) or a short hairpin RNA directed against CXCR4 (CXCR4 shRNA). CXCR4 expression declined with increasing viral mulitplicity of infection (MOI). (**B**) Animals were injected with U87 cells expressing either control (scrambled, sc-U87-Luc) or a CXCR4 specific shRNA (shCXCR4-U87-Luc). Growth curves were derived from serial bioluminesence imaging measurements (six to eight animals per experimental group) over the four-week experimental period post tumor cell implantation. Presented are the mean Bioluminescence Ratios (photon flux week (1–4)/photon flux hr48) ± SEM for each group. ^*^ = *P*<0.005 as determined by two-way ANOVA.

We previously demonstrated that systemic administration of the specific CXCR4 antagonist AMD 3100, inhibited the intracranial growth of U87 glioblastoma xenografts by increasing apoptosis and decreasing proliferation of tumor cells [14]. Both tumor cells and endothelial cells express CXCR4, and to distinguish whether tumor cell-CXCR4 function is required for tumor growth, we depleted CXCR4 by shRNA-mediated knock-down in U87 glioblastoma cells that had also been engineered to express a fusion protein of firefly luciferase and eGFP (shCXCR4-U87-Luc). Control cells were generated through expression of a scrambled shRNA (sc-U87-Luc). CXCR4 depletion was confirmed by western blot analysis (**Figure 1A**). Intracranial xenografts of shCXCR4-U87-Luc and sc-U87-Luc cells were generated in nude mice as described [26], [31]. Bioluminescence imaging 48 hrs post-intracranial injection was similar between the two groups [mean photon flux for sc-U87-Luc: 6.78×10^6^; and for shCXCR4-U87-Luc: 7.17×10^6^] suggesting that CXCR4 was not required for tumor cell engraftment. In contrast, CXCR4 depletion in U87 cells significantly suppressed their intracranial growth over a four-week experimental period (**Figure 1B**). These data strongly indicate that tumor cell CXCR4 function is required for tumor growth.

**Figure 2:**
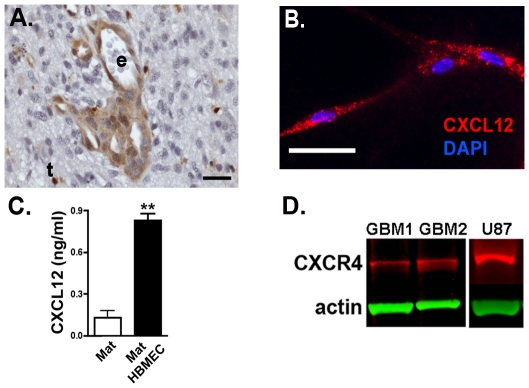
Primary human brain endothelial cells express CXCL12 and GBM cells express CXCR4. (**A**) Human glioblastoma specimen immunostained for CXCL12 (brown) demonstrates expression in vascular endothelial cells. t = tumor cells, e = cross-section through tumor-associated capillary, and Scale bar = 25 µm. (**B**) HBMECs, in co-culture on Matrigel, express CXCL12 (red). Nuclei are counterstained blue with DAPI. Scale bar equals 25 µm. (**C**) Human brain micro-vascular endothelial cells cultured on Matrigel (Mat HBMEC) secrete CXCL12 into the media as determined by ELISA. Mat alone indicates results from Matrigel alone-conditioned media. N = 3. ** = p<0.005 as determined by two-tailed *t*-test. (**D**) Single cell suspensions from two different adult GBM patients (GBM1, GBM2) and cultured U87 cells express CXCR4 by western blotting. CXCR4 appears red and the actin loading control appears green.

*In vivo*, CXCR4 function depends upon its ligand CXCL12. As previously described [11], [14], [15], [17], [39] and illustrated in **Figure 2A**, CXCL12 is localized to the endothelium of tumor-associated blood vessels in GBM. Therefore, we hypothesized that similar to normal neural stem cell niches [40], endothelial cell CXCL12 and tumor cell CXCR4 would play important roles in the biology of the GBM perivascular stem-like cell niche.

To assess whether CXCR4 mediates direct interactions between GBM and endothelial cells we turned to an *in vitro* co-culture model similar to that used by others [8], [16], in which primary human brain microvascular endothelial cells (HBMECs) and either U87 cells, or primary GBM cell isolates were cultured together in extracellular matrix (Matrigel). While the mouse sarcoma origin of Matrigel could limit its relevance in modeling the brain perivascular space, the primary components of Matrigel, including laminin, heparan sulfate proteoglycans, collagen IV and nidogen [41], are known to be essential components of brain germinal matrices, as well as the subendothelial cell basement membrane of the brain microvasculature [42]. The appropriateness of Matrigel for these studies is further supported by Matrigel's successful application in studies of neural stem cells [43], [44], [45] and human brain tumor cells [6].

When cultured in standard fashion on tissue culture plastic, HBMECs grow as a monolayer in which many individual cells assume an “epithelioid” morphology with abundant cytoplasm surrounding a round nucleus (**Figure S1A**). In contrast, when plated on Matrigel, HBMECs adopt a lattice-like configuration reminiscent of a capillary network in which individual cells exhibit a more native morphology characterized by an elongated nucleus and cell body (**Figure S1B**). Reproducible lattice networks were not observed when HBMECs were cultured on plastic, glass, fibronectin or gelatin (data not shown). This restricted distribution of HBMECs in Matrigel better models the arrangement of HBMECs *in vivo* when compared to the uniform distribution of cells when HBMEC were cultured as a monolayer on plastic.

To determine whether HBMECs cultured in Matrigel express CXCL12, we performed immunofluorescence labeling of fixed HBMECs (**Figure 2B**) and CXCL12 ELISAs on supernatants collected from HBMEC cultures (**Figure 2C**). We found that CXCL12 protein was present in HBMEC cells and released to the culture media. Thus, similar to native GBM vasculature, HBMEC Matrigel cultures could provide CXCL12 in a spatially restricted manner. Consistent with prior reports, U87 cells and primary GBM cell isolates express CXCR4 (**Figure 2D**).

**Figure 3:**
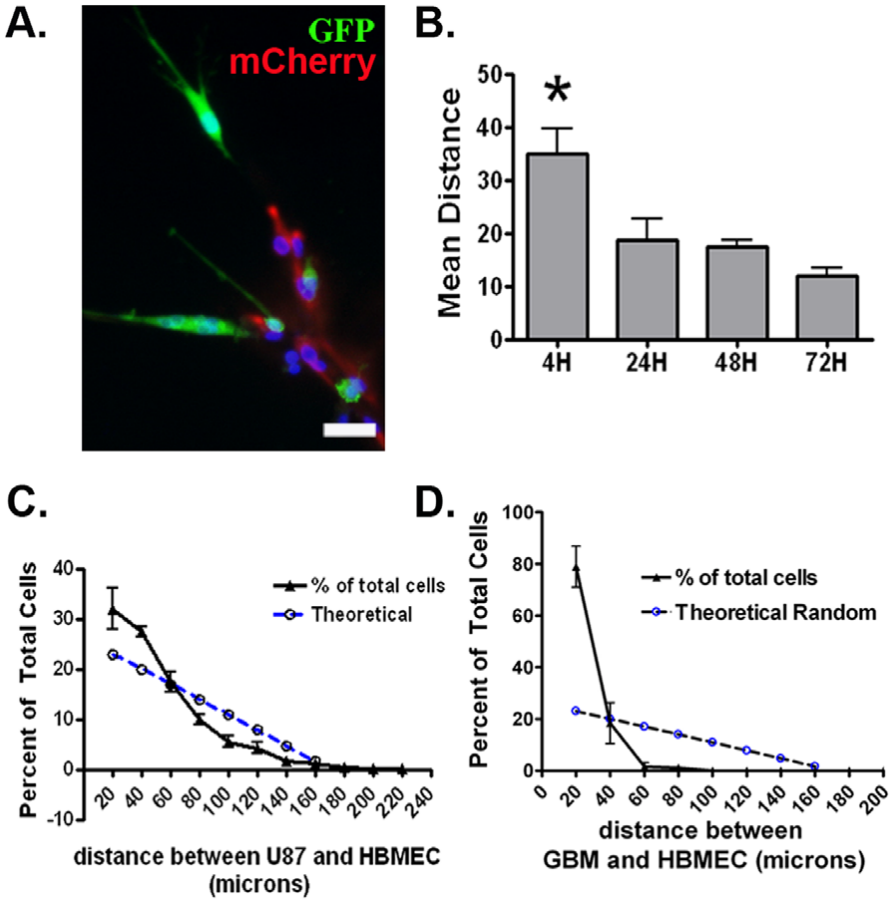
U87 and primary GBM cells localize to the peri-endothelial domain when plated with HBMECs on Matrigel. (**A**) Twenty-four hours after establishing a capillary-like network of mcherry-expressing HBMECs in Matrigel, eGFP-expressing U87 cells were added to the culture. Within 24 hours U87 cells were seen in physical contact with HBMECs. Scale bar = 50 microns. (**B**) The mean distances between U87 cells (500 to 800 cells) and HBMECs were calculated at different time points after the addition of the tumor cells to the HBMEC networks. There was a significant increase in co-localization (reduction in mean distance) within 24 hrs, which was maintained over a 72 hr period. * = p<0.05 as determined by one way ANOVA for the means of three separate experiments involving 500–800 measurements per experiment. (**C**) The distance between approximately 1000 eGFP-expressing U87 cells and mCherry fluorescent protein-expressing HBMECs in co-culture (24 hrs) was measured and the distribution was plotted as the percentage of total cells in 20 micron increments (black triangles). More than 50% of the total U87 cells in the culture were within 40 microns of an endothelial cell. A theoretical plot of a random distribution of cells is shown (open circles). (**D**) The distance between GFAP positive GBM cells and mCherry fluorescent protein-expressing HBMECs in co-culture were measured and the distribution was plotted as the percentage of total cells in 20 micron increments (black triangles). Error bars represent SEM from three independent experiments involving three different GBM isolates. Approximately 500 GBM (GFAP positive) cells were counted. Nearly 80% of the GFAP positive GBM cells in the culture were within 20 microns of an endothelial cell. A theoretical plot of a random distribution of cells is shown (open circles).

To ascertain whether HBMEC-derived CXCL12 would influence the behavior of GBM cells we first sought to determine whether HBMECs in this capillary-like configuration impose a spatial organization to the culture milieu. U87-Luc cells were added to a preformed HBMEC network in which the endothelial cells had been engineered to express mCherry fluorescent protein. U87 cells appeared to localize to the peri-endothelial cell space and to make direct contact with endothelial cells (**Figure 3A**). Co-localization of most U87 cells to the HBMECs occurred within the first 24 hours and the mean distance between tumor and endothelial cells did not significantly change beyond that time (**Figure 3B**). Hence, for all subsequent co-localization experiments measurements were performed after 24 hrs of addition of tumor cells. To more quantitatively evaluate this co-localization we analyzed the data as follows. The spaces within the lattice-work approximate circular elements. We determined that the mean radius of these elements was 155 µm. By calculating the fractional area of concentric circles within each space, we were able to derive a relation between distance and cell number for a *theoretical random* distribution of cells within the lattice-work (**Figure S2A**). By measuring the mean distance between eGFP-expressing U87 cells and mCherry-expressing HBMECs (**Figure S2A–D**), we found that U87 cells were not distributed randomly within the culture dish but instead were clustered around endothelial cells. Approximately 50% of U87 cells were localized within 40 µm of an endothelial cell with approximately 30% of U87 nuclei within 20 µm of an endothelial cell, including cells in physical contact with the endothelium (**Figure 2C**). The non-random nature of this distribution indicated that U87 cells were co-localized with HBMECs.

To determine whether primary GBM cells would also localize to the peri-endothelial cell space, we added primary GBM cells from patient specimens to pre-formed HBMEC lattice/tubule structures. Primary GBM tumor cells were identified by their expression of the standard histopathological marker for astrocytoma (glioma) cells, GFAP [46]. We found that primary GBM cells also co-localized with the endothelial cells (**Figure S3A**) and made direct cell-to-cell contacts via their processes (**Figure S3B**).

Primary GBM cells were also not distributed randomly within the culture dish but instead were clustered around endothelial cells (**Figure 3D**). The distance between individual endothelial cells and greater than 500 GBM cells, derived from independent experiments utilizing three different primary isolates, was measured. Nearly 80% of GBM cells were localized within 20 µm of an endothelial cell. The non-random nature of this distribution indicated that primary GBM cells were also co-localized with HBMECs.

**Figure 4:**
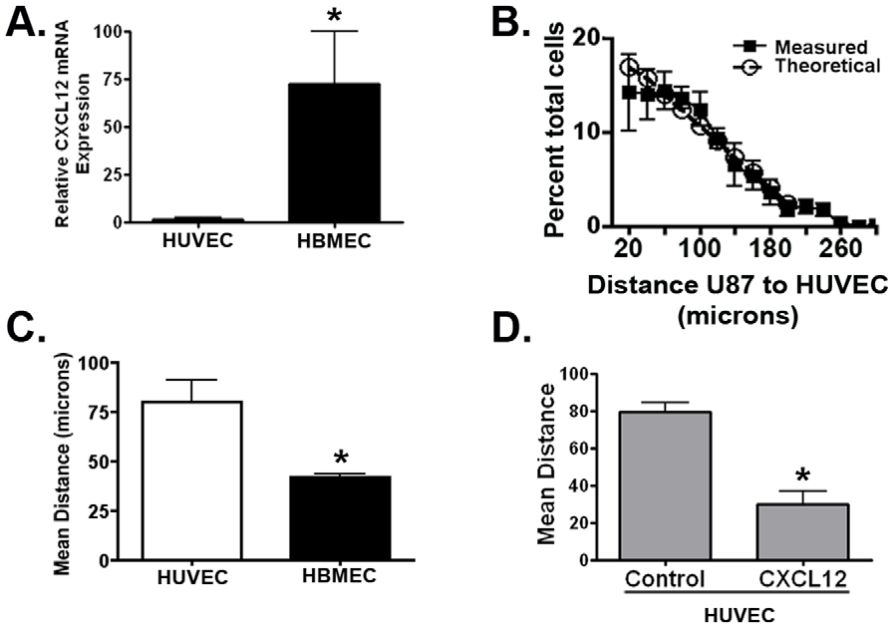
U87 localization to the peri-endothelial space is correlated with CXCL12 expression. (**A**) Quantitative PCR reveals that HUVECs express significantly lower levels of CXCL12 than HBMECs. N = 3, ** = p<0.005 as determined by two-tailed *t*-test. (**B**) The distance between greater than 1000 eGFP-expressing U87 cells and mCherry fluorescent protein-expressing HUVECs in co-culture was measured and the distribution was plotted as the percentage of total cells in 20 micron increments (filled squares). Approximately 30% of the total U87 cells in the culture were within 40 microns of an endothelial cell (compare to **Figure 3B**). This distribution does not differ from a theoretical plot of a random distribution of cells (open circles) (**C**) A statistically significant difference existed between the mean distance of U87 cells to HBMECs versus the mean difference between U87 and HUVECs. * = p<0.05 as determined by two-tailed *t*-test for the means of four separate experiments involving approximately 330 measurements per endothelial co-culture per experiment. (**D**) When cultured with HUVECs that are engineered to over-express CXCL12, U87s show a similar co-localization pattern (as seen with HBMECs) consistent with a significant reduction in mean distance when compared to co-cultures with control HUVECs. * = p<0.05 as determined by two-tailed *t*-test for the means of two separate experiments involving approximately 300 measurements per endothelial co-culture per experiment.

Prior studies have shown that CXCR4 activation induces chemotaxis and survival in U87 cells [14]. Given the recognized role that the CXCL12-CXCR4 pathway plays in chemo-attraction of multiple cell types (lymphocytes, neurons, tumor cells [47]), we evaluated the hypothesis that GBM cells are localized to the peri-endothelial cell domain in a CXCL12-dependent fashion. We first cultured U87 cells with human umbilical vein endothelial cells (HUVECs), which form similar capillary-like networks in Matrigel but express significantly lower levels of CXCL12 mRNA (**Figure 4A**) and protein (**Figure S4A**) compared to HBMECs. Consistent with a dose-dependent CXCL12 effect on U87 localization to the peri-endothelial cell space, U87 cells were distributed in a random fashion within the HUVEC lattice with fewer U87 cells in close apposition to HUVECs compared to HBMECs (compare **Figure 4B** to **F** **igure 3C**). Consequently the mean distance between U87 and HUVECs was significantly higher compared to that of HBMECs (**Figure 4C**). To determine whether CXCL12 expression was sufficient to endow HUVECs with a localizing effect similar to HBMECs, we engineered HUVECs to express CXCL12. HUVEC-CXCL12 cells release comparable levels of CXCL12 into the culture media as HBMECs (**Figure S4A**). Co-culture of U87s with HUVEC-CXCL12 cells resulted in localization of U87cells to the peri-endothelial cell space (**Figure S4B, C**). The mean distance between U87 and HUVEC-CXCL12 cells was similar to that of HBMECs (**Figure 4D**). These data indicate that endothelial cell secretion of CXCL12 is sufficient to induce the localization of CXCR4-expressing GBM cells.

**Figure 5:**
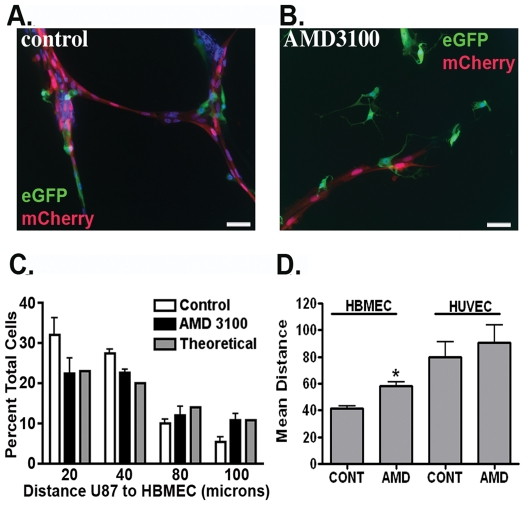
U87 localization to the peri-endothelial space is blocked by the CXCR4 antagonist AMD 3100. (**A**) GFP-expressing U87 cells (green) are localized to mCherry-expressing HBMECs (red). (**B**) Treatment of parallel co-cultures as in **A** with AMD 3100 results in failure of U87 cells (green) to make consistent contact with endothelial cells (red). Scale bar equals 25 microns. (**C**) The CXCR4 antagonist AMD 3100 redistributes U87 cells, decreasing the number of cells within the nearest proximity to HBMECs and increasing the number of cells at greater distance. * = p<0.05 as determined by two-way ANOVA. (**D**) AMD 3100 increased the mean distance between U87 cells and HBMECs but had no effect on the mean distance between U87 cells and HUVECs. * = p<0.05 as determined by two-tailed *t*-test.

To determine whether CXCR4 activation was necessary for the co-localization of GBM cells with endothelial cells we treated U87-HBMEC cultures with AMD3100. This resulted in the dispersal of U87 cells from the peri-endothelial cell domain (**Figure 5A and 5B**). AMD3100 reduced the fraction of U87 cells within 40 microns of an endothelial cell (**Figure 5C**), and the mean distance between U87 and HBMECs was increased from 40 to nearly 60 µm in these experiments (**Figure 5D**). The resulting distribution of U87 cells in AMD3100 treated cultures was no longer different from the random distribution. As an additional control, we evaluated the effects of AMD3100 on the mean distance between U87 and HUVECs and found that in the absence of CXCL12 secretion, AMD3100 had no effect on U87 cell distribution (**Figure 5D**). Together, the above data suggested that endothelial cell-derived CXCL12 induced chemo-localization of U87 cells to the peri-endothelial cell domain in a CXCR4 dependent fashion.

**Figure 6:**
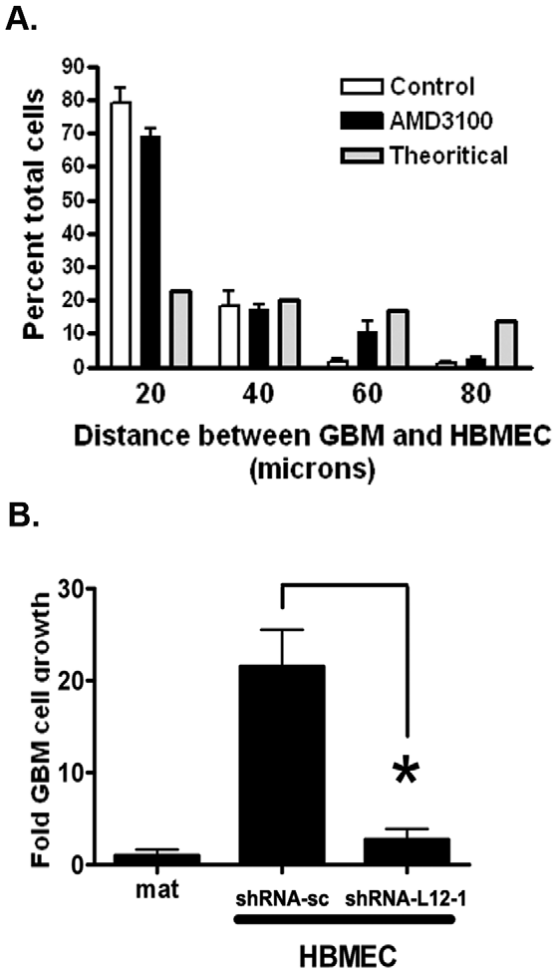
The chemotactic and trophic effect of the peri-endothelial space on primary GBMs is blocked by pharmacologic or genetic inhibition of the CXCL12-CXCR4 pathway. (**A**) The distance between GFAP positive GBM cells and mCherry fluorescent protein-expressing HBMECs in co-culture was measured as in Figure 3. Error bars represent SEM from three independent experiments involving three different GBM isolates. Approximately 100 GBM (GFAP positive) cells were counted in each condition. Nearly 80% of the GFAP positive GBM cells in the culture were within 20 microns of an endothelial cell. Treatment with AMD3100 resulted in a reduction in the proportion of GBM cells in closest proximity to endothelial cells and an increase in the proportion of cells at greater distances. P<0.005 by two-way ANOVA. (**B**) Primary GBM cells co-cultured with HBMECs expressing a scrambled shRNA (sh-RNA-sc) show increased growth after 72 hours in culture, as measured by number of GFAP-positive tumor cells, compared to growth on Matrigel alone. The trophic effect of HBMECs on primary GBM cells is abrogated upon depletion of CXCL12 expression. N = 3, * = P<0.005 as determined by one-way ANOVA with Dunnett's post-test for multiple comparisons.

We next sought to determine whether inhibiting CXCR4 would also alter the localization of primary GBM cells to the peri-endothelial cell space. We treated three different primary HBMEC-GBM co-cultures with AMD3100 and performed the same localization analysis as described above. Similar to the results presented in **Figure 2**, nearly 80% of primary GBM cells were localized within 20 microns of an endothelial cell in the untreated control condition (**Figure 6A**). Treatment with AMD3100 (2.5 ng/ml, 24 h) resulted in a modest, but consistent and significant (P<0.005, two-way ANOVA), change in GBM cell distribution. Notably there was a decrease in the proportion of cells within 20 microns of an endothelial cell (77+/−0.33% vs. 69+/−3%) and a commensurate increase in the proportion of GBM cells at greater distances from the peri-endothelial cell domain. The small but reproducible effect of CXCR4 antagonism on GBM cell localization suggests that the CXCL12-CXCR4 pathway participates in localization of primary GBM cells to the peri-endothelial cell domain but does not account for all of the chemo-localization effect of endothelial cells on GBM cells.

Among the implications of peri-endothelial localization is the potential for trophic interactions between endothelial cells and GBM cells. To evaluate this possibility, U87 cells or primary GBM specimens were added to HBMEC networks and cell number was measured using bioluminescence imaging (for U87) or the number of GFAP positive cells/well (for primary GBM) and quantified using a computer-controlled epi-fluorescence microscope and automated image analysis. Consistent with endothelial cells providing trophic support for GBM cells, co-culture of either U87 or primary GBM cell isolates with HBMECs in Matrigel resulted in increased GBM cell number (**Figure S5A–B**). The magnitude of the trophic effect ranged between 1.5 and greater than twenty fold for the different primary GBM specimens when compared to GBM cells grown on Matrigel alone.

To determine whether this trophic effect was mediated by CXCL12, we generated lentivirus-encoding shRNA directed against CXCL12, or encoding a scrambled shRNA control. CXCL12 mRNA and protein expression was reduced in primary HBMECs infected with either of two shRNAs directed against CXCL12, but not in HBMECs infected with lentivirus encoding the scrambled shRNA control (**Figures S4 and S6**). Hairpin shRNA-L12-1 reduced CXCL12 expression to a significantly lower level than shRNA-L12-2 and thus, only shRNA-L12-1 was used for subsequent evaluations of the effect of reduced endothelial cell CXCL12 expression on primary GBM growth.

Targeted reduction of CXCL12 expression with lentivirus encoding shRNA had no effect on endothelial lattice formation in Matrigel (data not shown) suggesting that CXCL12 is not required for this process *in vitro*. HBMECs infected with lentivirus encoding scrambled shRNA induced a nearly twenty-fold increase in GBM cell number compared to GBM mono-culture. Despite consistent lattice formation, reduced expression of CXCL12 in HBMECs completely abrogated the trophic effect of endothelial cells on primary GBM cell growth (**Figure 6B**). Concordantly, culturing of U87 GBM cells with HUVECs did not enhance tumor cell growth (data not shown). Together, these data indicated that endothelial cell-derived CXCL12 mediates a direct trophic effect of endothelial cells on GBMs cells.

To determine whether CXCL12 effects were mediated by CXCR4, we treated cultures with AMD3100 and measured tumor cell growth by bioluminescence imaging. The trophic effect of endothelial cells on primary GBM cells was inhibited by the specific CXCR4 antagonist, AMD3100 (**Figure S5A**). Consistent with prior studies in which AMD3100 and a second specific CXCR4 antagonist AMD3465 only blocked CXCL12-induced U87 cell growth [14], [31], AMD3100 only inhibited primary GBM cell growth when the GBM cells were co-cultured with HBMECs, but not when they were in mono-culture. These data suggest that endothelial cells generated CXCL12 is responsible for the CXCR4 mediated trophic effect on GBM cells. In accordance with this conclusion, and consistent with published *in vivo* effects [14], AMD3100 treatment increased GBM cell apoptosis as measured by TUNEL staining (**Figure S5B**).

Recently, a second CXCL12 receptor, CXCR7, has been reported to mediate survival effects in certain glioma cell lines [34]. To determine whether CXCR7 mediates GBM cell survival in response to endothelial cell-derived CXCL12 we evaluated CXCR7 expression in U87-HBMEC co-cultures by immunohistochemistry. Consistent with prior reports [48], [49], HBMECs demonstrated a membranous pattern of CXCR7 expression cells (**Figure S7A**). In contrast, U87 cells exhibited little to no CXCR7 expression. To evaluate whether CXCR7 might also contribute to the trophic effect of endothelial cells we compared the effects of antagonizing both CXCL12 receptors CXCR4 and CXCR7 on U87 cell growth. Inhibition of CXCR4 by AMD3100 completely blocked HBMEC-induced enhanced growth. In contrast, the effects of the selective CXCR7 antagonists CCX733 and CCX771 were indistinguishable from the inactive control compound CCX704 (**Figure S7B**). Together these data suggest that in our co-culture models, endothelial cell-derived CXCL12 exerts a chemo-localization and a trophic effect on GBM cells through its primary signaling receptor CXCR4.

## Discussion

There is an urgent need to understand the biology of microvascular endothelial cell-GBM interactions since they appear to support tumor growth, spread and resistance to treatment [6], [7], [16], [39]. Practical experimental systems will be critical for establishing the basic biology and for high throughput screening for drugs capable of disrupting this interaction. The co-cultures described here are a tractable and relevant experimental system, as they recapitulate the chemo-localization and trophic effects of human brain endothelial cells on primary GBM cells. This simplified system is optimally suited for the detailed quantification of tumor cell migration as well as the trophic effects resulting from specific cell-cell interactions. In addition, uniform oxygenation in the co-cultures allows experimental separation of the trophic functions of endothelial cells away from their vascular functions such as oxygenation. Further, the absence of vasculogenesis in the co-cultures successfully isolates the contributions of CXCL12/CXCR4 to growth regulation from their role in blood vessel formation. The ability to distinguish between these functions will be critical in interpreting and translating findings such as the correlation between increased CXCL12 signaling and increased vasculogenesis in an irradiated model of GBM xenografts [33].

Using the co-culture system, we identified CXCL12 as a mediator of endothelial cell trophic functions. Similar to its effects within germinal niches like the bone marrow [19], [50], [51], [52] and external granule cell layer of the developing cerebellum [22], CXCL12 chemo-attracts brain tumor cells to the peri-endothelial cell space and stimulates their growth within that domain. The ability to target these endothelial cell functions could have significant therapeutic implications. The peri-endothelial cell space has been proposed to be a specialized brain tumor stem-like cell niche [6], [53] as well as a route for GBM invasion [3], [12], [16], [39]. While the existence of a distinct stem-like cell sub-population in GBM is controversial [54], tumor-derived cells with enhanced tumor-initiating activity have been repeatedly described (reviewed in [55], [56]. Among the properties of these specialized cells appears to be enhanced resistance to radiation and chemotherapy [7], [9], [57]. If resistance to radiation therapy and chemotherapy are dependent upon localization to the peri-endothelial cell space, then mobilization of brain tumor cells with CXCR4 antagonists could be used in combination with radiation or standard chemotherapy to greater effect. This mechanism could in fact be the basis for the synergy observed between AMD3100 and BCNU in the treatment of intracranial U87 xenografts [58]. Similar observations were recently reported for acute myelogenous leukemia in which antagonism of CXCL12 resulted in leukemic blast mobilization from the bone marrow and enhanced anti-leukemia effect of tyrosine kinase inhibitors [59]. The combination of AMD3100 as a mobilizing agent for leukemic blasts and standard chemotherapy with mitoxanthrone, etoposide and cytarabine for the treatment of patients with relapsed or refractory AML is currently being evaluated in clinical trials.

Further, it has been shown that CXCR4 mediates the perivascular migration of GBM cells [16], [39]. The finding that tumor-associated endothelial cells express high levels of CXCL12 [13], [14], [39] supported the hypothesis that this characteristic feature of GBM would involve CXCR4. The present findings advance this hypothesis and provide additional rationale for pursuing CXCR4 antagonism in the treatment of GBM. Of particular importance may be the combination of CXCR4 antagonism and anti-angiogenic therapy. Anti-angiogenic therapy may decrease the number of perivascular sites capable of supporting and protecting brain tumor cells. Thus, combination therapy could provide both a structural and functional disruption of the perivasclar niche.

In summary, primary GBM and HBMEC co-culture has identified an important role for CXCL12 in cell-cell interactions between GBM cells and endothelial cells. These data suggest that endothelial cells have “extra-vascular” functions that can directly drive tumor growth. Whether endothelial cell-derived molecules like CXCL12 create a specialized niche in the perivascular space, and how these molecules relate to the phenotype of stem-like cells remains to be determined. What is clear is that targeting both angiogenesis and endothelial cell function may represent a novel and powerful approach in the treatment of malignant brain tumors.

## Supporting Information

Figure S1 **Primary HBMECs Form Capillary-Like Networks in Matrigel.** (**A**) Primary HBMECs grow as a monolayer culture on uncoated coverslips (or plates) in endothelial cell growth media for 24 hours. Scale bar equals 50 µm. (**B**) HBMECs plated at similar density to (**A**), 30 minutes after establishing a Matrigel layer within the culture dish, become organized into a capillary-like network. Scale bar equals 100 µm.
(TIF)

Figure S2 **Localization of GBM cells to the Perivascular space.** (**A**) Algorithm for calculating *random* distribution of tumor cells within HBMEC lattice. The lattice-work is assumed to contain concentric circles whose fractional area is calculated as shown. Fractional area is graphed as a function of distance from endothelial cells (ECs) to determine how a random distribution of tumor cells would appear. Red and gray bars on graph correspond to red and gray donuts in cartoon. (**B**) A low magnification image of an HBMEC lattice. (**C**) Tumor cells (green) localize to HBMEC (red). (**D**) The distance between tumor cell nuclei and the nearest endothelial cell body was measured using Axiovision software (Zeiss).
(TIF)

Figure S3 **Primary GBM cells make direct contacts with HBMECs** ***in vitro*** **.** (**A**) Twenty-four hours after establishing a capillary-like network of mCherry-expressing HBMECs in Matrigel, primary GBM cell isolates collected from three different patients were added to the culture. 24 hours later, GFAP-positive GBM cells were seen in physical contact with HBMECs. (**B**) A GFAP positive GBM cell (green) extends a process to contact an HBMEC (*). Also note the GFAP- negative GBM-derived cells, identified by nuclear DAPI (blue) staining only, at a distance from the mCherry expressing HBMEC cells. Scale bar = 50 µm in panel A and 25 µm in panel D.
(TIF)

Figure S4 **Manipulation of CXCL12 expression regulates endothelial cell function.** (**A**) The amount of CXCL12 secreted into the media by HUVECs (control or CXCL12 over-expressing) and HBMECs (control or infected with CXCL12 shRNAs) were quantified using ELISA. CXCL12 over-expression of HUVECs significantly increased the amount of chemokine secreted to the media (*p<0.05, as determined by one-way ANOVA and Newman-Keuls multiple comparison test). Supernatant from HBMECs contained significantly higher amounts of CXCL12 compared to HUVECs (#p<0.05, as determined by one-way ANOVA and Newman-Keuls multiple comparison test). CXCL12 knockdown reduced supernatant CXCL12 levels in HBMECs. (**B**) U87 cells (expressing eGFP) preferentially colocalize with CXCL12 over-expressing but not control (**C**) HUVECs (expressing mCherry). Scale bar = 500 µm.
(TIF)

Figure S5 **Primary HBMEC exert a trophic effect on primary GBM cells that is blocked by AMD3100.** (**A**) The trophic effect of endothelial cells on luciferase-expressing U87 cells was measured in four separate experiments by bioluminescence imaging (BLI) of U87 luciferase activity after 3 days in co-culture. In the absence of HBMECs, AMD3100 has no effect on total cell number as measured by BLI. Co-culture with endothelial cells results in a significant increase in BLI and this effect was blocked by both AMD3100. P<0.05 as determined by one-way ANOVA with Dunnett's post-test for multiple comparisons. (**B**) Co-culture with HBMEC in Matrigel (Mat) stimulates primary adult GBM cell growth at 72 hours. The trophic effect is inhibited by AMD3100 (AMD). Shown are the means and SEM of values normalized from triplicate cultures involving a single primary GBM isolate. ** = P<0.005 as determined by one-way ANOVA with Dunnett's post-test for multiple comparisons. Similar results were obtained with two other primary GBM cell isolates. (**C**) TUNEL assay in parallel primary GBM- endothelial cell co-cultures as those described in (**B**) indicates that treatment with AMD3100 increased GBM apoptosis (n = 3).
(TIF)

Figure S6 **Lentiviruses encoding shRNA targeting CXCL12 (shRNA-L12-1, 2) decrease CXCL12 mRNA levels in primary HBMEC cells relative to HBMECs infected with lentivirus encoding a scrambled control shRNA (shRNA-sc).** * = P<0.05 as determined by two-way Student's T-test, n = 3.
(TIF)

Figure S7 **Trophic effects of endothelial cell-derived CXCL12 are mediated through CXCR4.** (**A**) CXCR7 expression in U87 GBM cells grown in co-culture with HBMECs (top panels) or monoculture (bottom panels) was evaluated by immunohistochemistry. IgG controls are shown on the left and specific CXCR7 immunolocalization is on the right. U87 cells express GFP and appear green. HBMECs exhibit a membranous pattern of CXCR7 expression (arrow). In contrast, U87 cells exhibit little or no CXCR7 expression. Scale bar = 50 µM. (**B**) U87 cell growth was also measured in the presence of CXCR7 antagonists, CCX773 and CCX771, or the inactive control compound CCX704. The effects of CCX733 and CCX771 were indistinguishable from the control compound CCX704.
(TIF)
