# Supplementary figures and images for: CXCL12 Mediates Trophic Interactions between Endothelial and Tumor Cells in Glioblastoma
Source: PLoS One. 2012 Mar 12;7(3):e33005. doi: 10.1371/journal.pone.0033005 (PMC3299723; doi:10.1371/journal.pone.0033005)

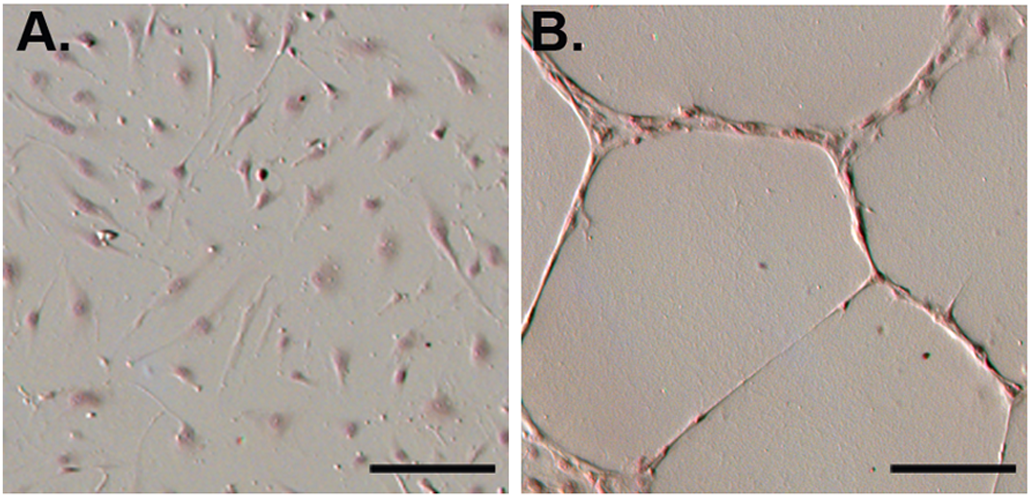

Supplement: Figure S1 — Primary HBMECs Form Capillary-Like Networks in Matrigel. (A) Primary HBMECs grow as a monolayer culture on uncoated coverslips (or plates) in endothelial cell growth media for 24 hours. Scale bar equals 50 µm. (B) HBMECs plated at similar density to (A), 30 minutes after establishing a Matrigel layer within the culture dish, become organized into a capillary-like network. Scale bar equals 100 µm. (TIF) [file pone.0033005.s001.tif]

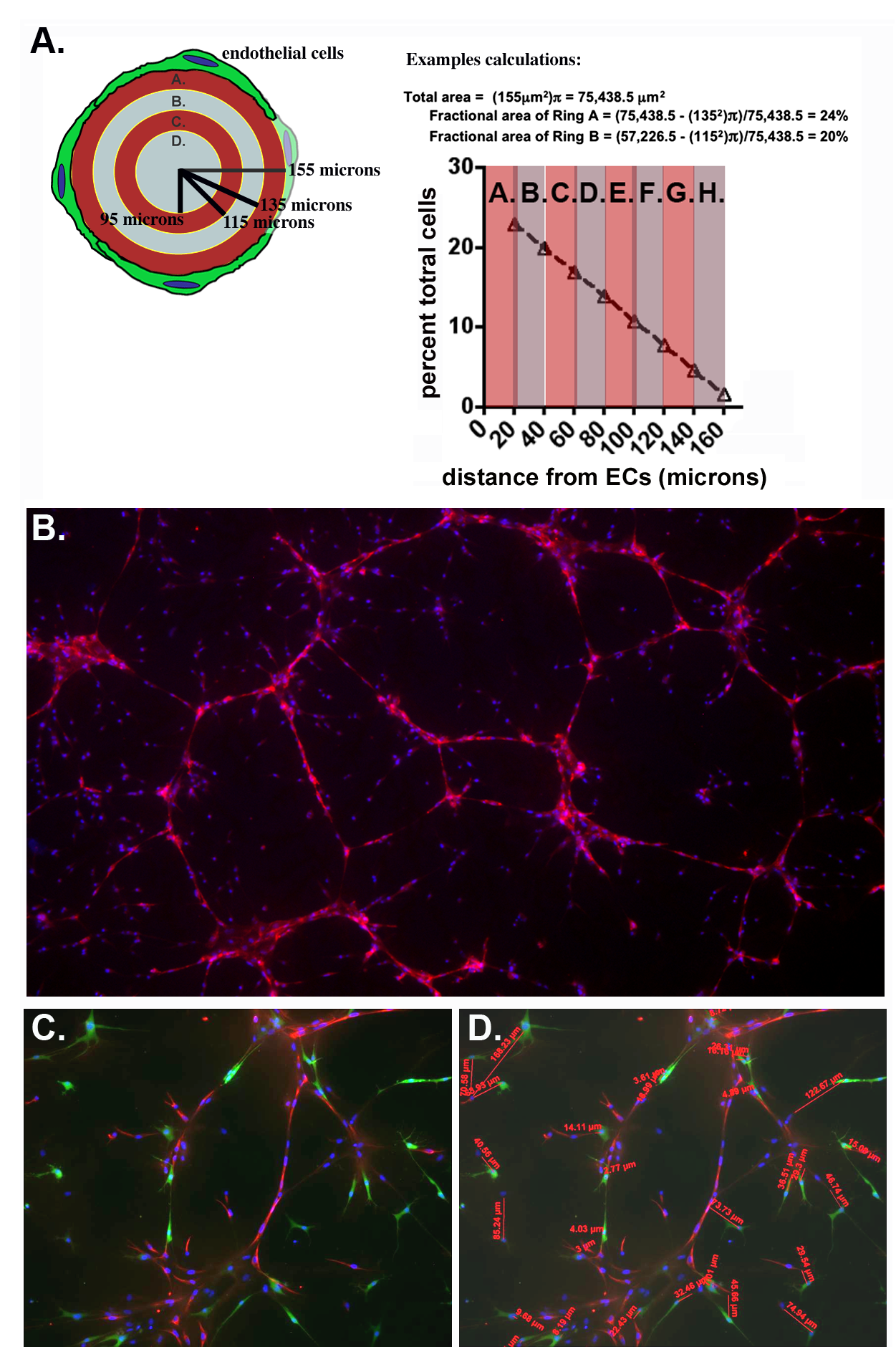

Supplement: Figure S2 — Localization of GBM cells to the Perivascular space. (A) Algorithm for calculating random distribution of tumor cells within HBMEC lattice. The lattice-work is assumed to contain concentric circles whose fractional area is calculated as shown. Fractional area is graphed as a function of distance from endothelial cells (ECs) to determine how a random distribution of tumor cells would appear. Red and gray bars on graph correspond to red and gray donuts in cartoon. (B) A low magnification image of an HBMEC lattice. (C) Tumor cells (green) localize to HBMEC (red). (D) The distance between tumor cell nuclei and the nearest endothelial cell body was measured using Axiovision software (Zeiss). (TIF) [file pone.0033005.s002.tif]

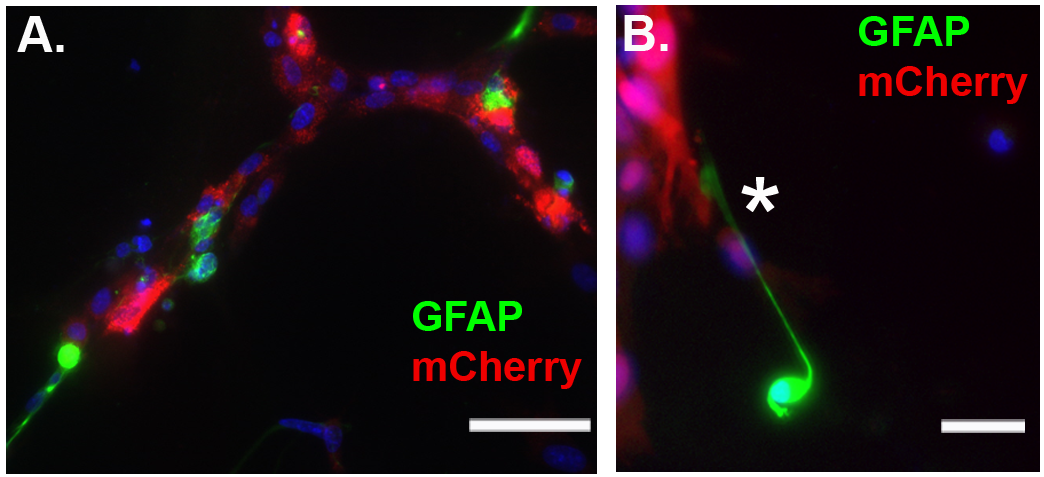

Supplement: Figure S3 — Primary GBM cells make direct contacts with HBMECs in vitro . (A) Twenty-four hours after establishing a capillary-like network of mCherry-expressing HBMECs in Matrigel, primary GBM cell isolates collected from three different patients were added to the culture. 24 hours later, GFAP-positive GBM cells were seen in physical contact with HBMECs. (B) A GFAP positive GBM cell (green) extends a process to contact an HBMEC (*). Also note the GFAP- negative GBM-derived cells, identified by nuclear DAPI (blue) staining only, at a distance from the mCherry expressing HBMEC cells. Scale bar = 50 µm in panel A and 25 µm in panel D. (TIF) [file pone.0033005.s003.tif]

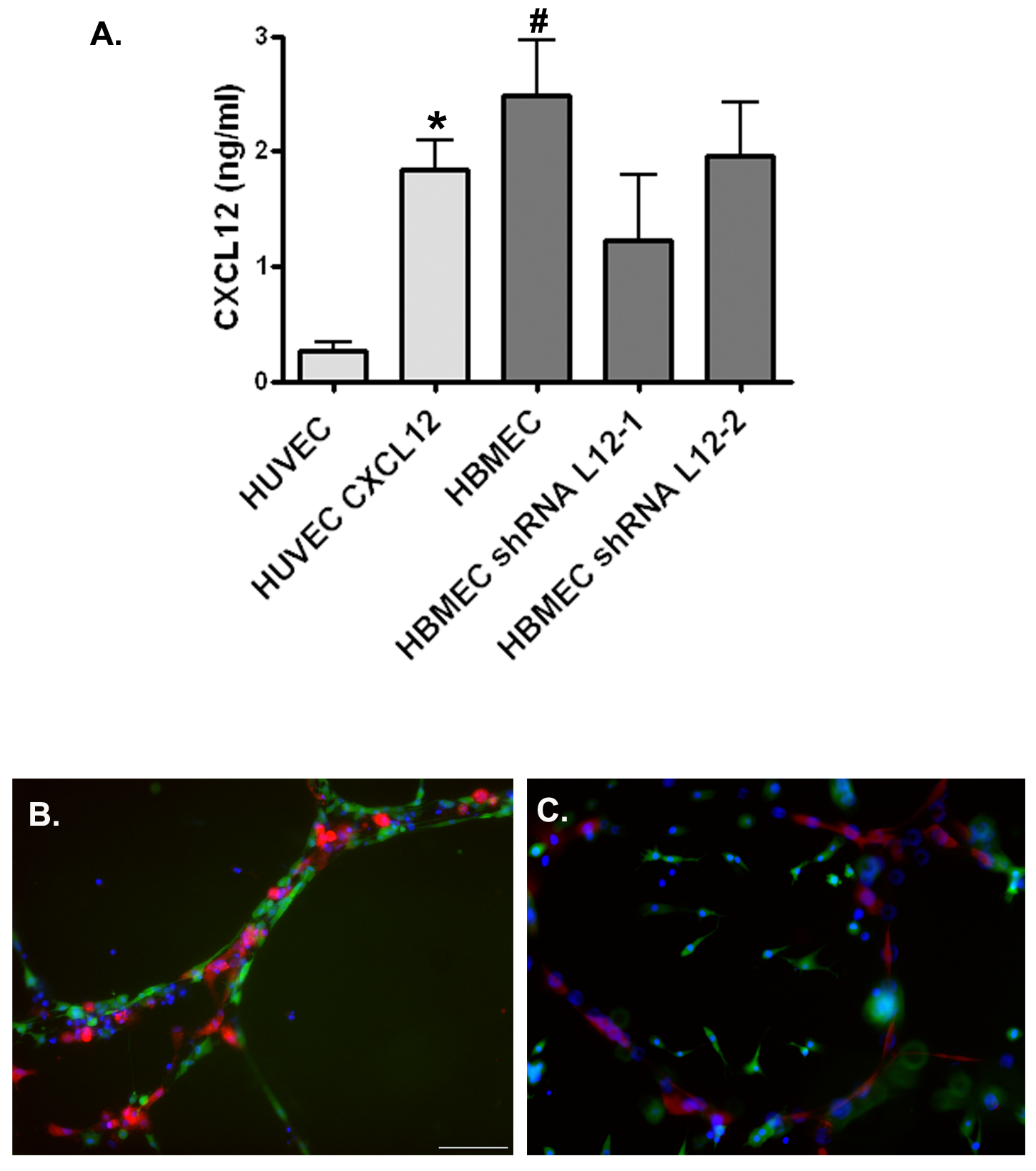

Supplement: Figure S4 — Manipulation of CXCL12 expression regulates endothelial cell function. (A) The amount of CXCL12 secreted into the media by HUVECs (control or CXCL12 over-expressing) and HBMECs (control or infected with CXCL12 shRNAs) were quantified using ELISA. CXCL12 over-expression of HUVECs significantly increased the amount of chemokine secreted to the media (*p<0.05, as determined by one-way ANOVA and Newman-Keuls multiple comparison test). Supernatant from HBMECs contained significantly higher amounts of CXCL12 compared to HUVECs (#p<0.05, as determined by one-way ANOVA and Newman-Keuls multiple comparison test). CXCL12 knockdown reduced supernatant CXCL12 levels in HBMECs. (B) U87 cells (expressing eGFP) preferentially colocalize with CXCL12 over-expressing but not control (C) HUVECs (expressing mCherry). Scale bar = 500 µm. (TIF) [file pone.0033005.s004.tif]

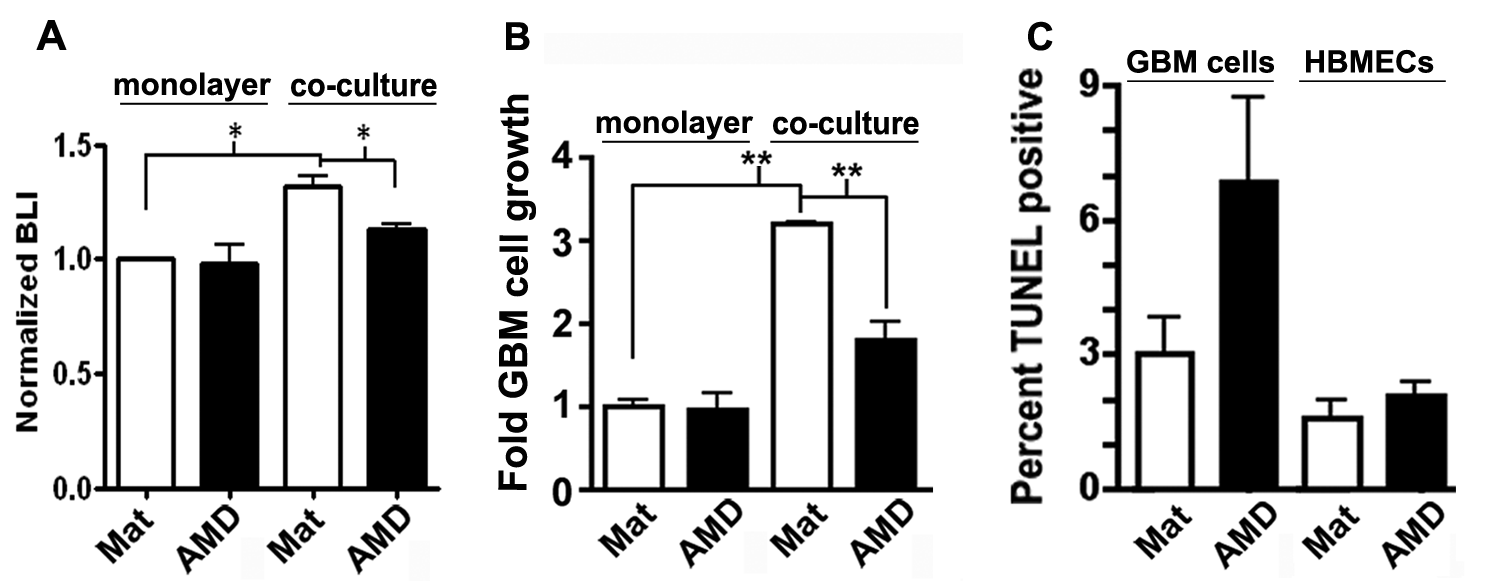

Supplement: Figure S5 — Primary HBMEC exert a trophic effect on primary GBM cells that is blocked by AMD3100. (A) The trophic effect of endothelial cells on luciferase-expressing U87 cells was measured in four separate experiments by bioluminescence imaging (BLI) of U87 luciferase activity after 3 days in co-culture. In the absence of HBMECs, AMD3100 has no effect on total cell number as measured by BLI. Co-culture with endothelial cells results in a significant increase in BLI and this effect was blocked by both AMD3100. P<0.05 as determined by one-way ANOVA with Dunnett's post-test for multiple comparisons. (B) Co-culture with HBMEC in Matrigel (Mat) stimulates primary adult GBM cell growth at 72 hours. The trophic effect is inhibited by AMD3100 (AMD). Shown are the means and SEM of values normalized from triplicate cultures involving a single primary GBM isolate. ** = P<0.005 as determined by one-way ANOVA with Dunnett's post-test for multiple comparisons. Similar results were obtained with two other primary GBM cell isolates. (C) TUNEL assay in parallel primary GBM- endothelial cell co-cultures as those described in (B) indicates that treatment with AMD3100 increased GBM apoptosis (n = 3). (TIF) [file pone.0033005.s005.tif]

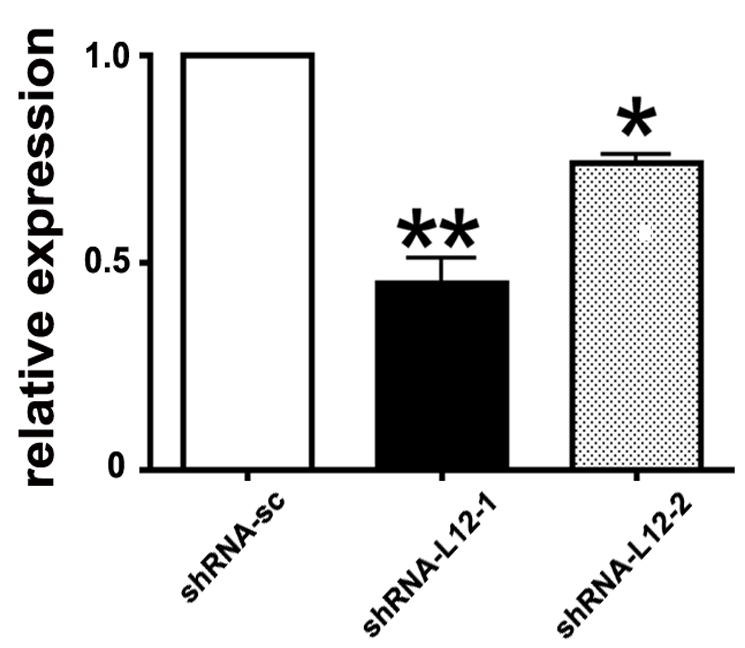

Supplement: Figure S6 — Lentiviruses encoding shRNA targeting CXCL12 (shRNA-L12-1, 2) decrease CXCL12 mRNA levels in primary HBMEC cells relative to HBMECs infected with lentivirus encoding a scrambled control shRNA (shRNA-sc). * = P<0.05 as determined by two-way Student's T-test, n = 3. (TIF) [file pone.0033005.s006.tif]

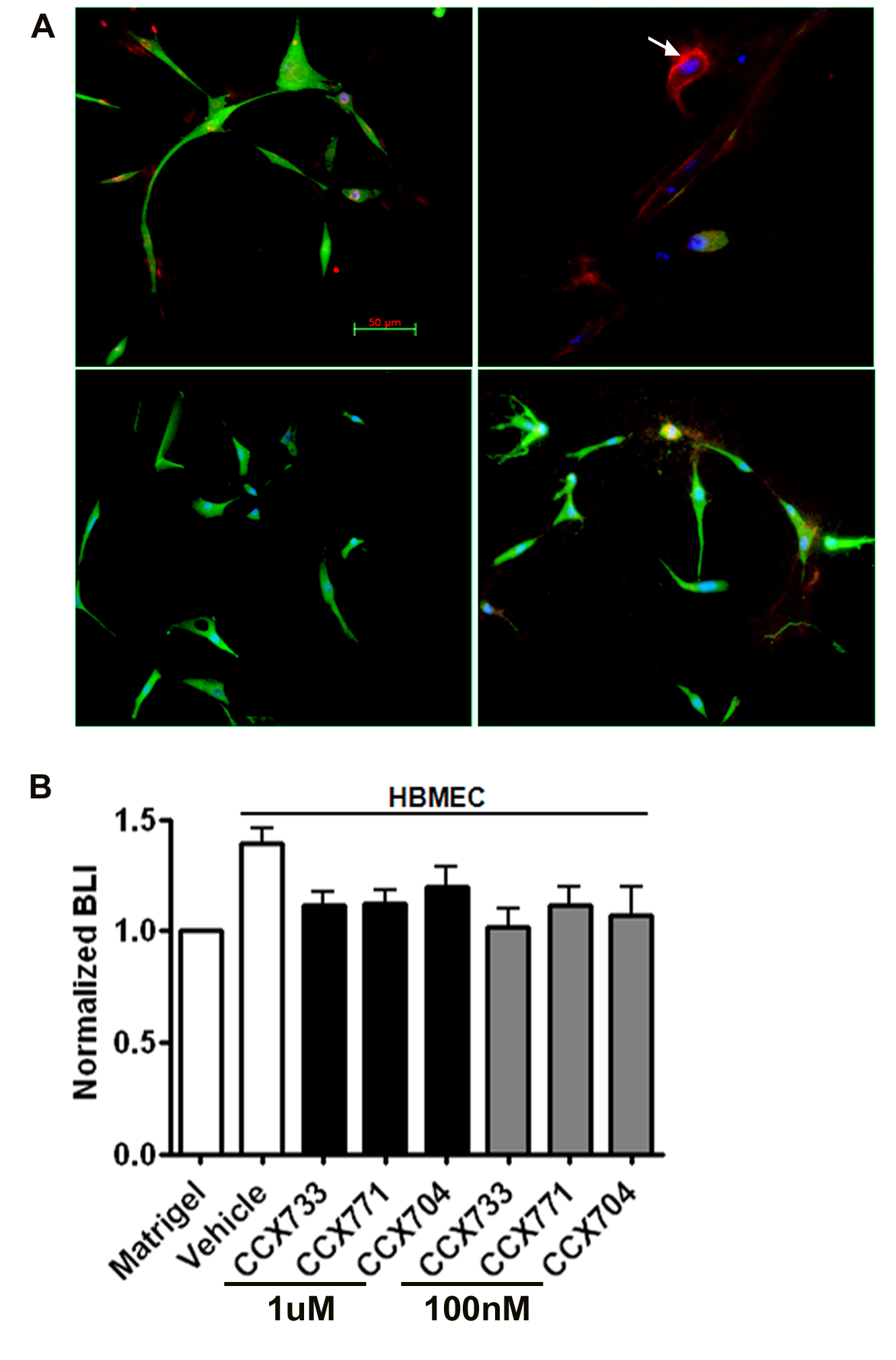

Supplement: Figure S7 — Trophic effects of endothelial cell-derived CXCL12 are mediated through CXCR4. (A) CXCR7 expression in U87 GBM cells grown in co-culture with HBMECs (top panels) or monoculture (bottom panels) was evaluated by immunohistochemistry. IgG controls are shown on the left and specific CXCR7 immunolocalization is on the right. U87 cells express GFP and appear green. HBMECs exhibit a membranous pattern of CXCR7 expression (arrow). In contrast, U87 cells exhibit little or no CXCR7 expression. Scale bar = 50 µM. (B) U87 cell growth was also measured in the presence of CXCR7 antagonists, CCX773 and CCX771, or the inactive control compound CCX704. The effects of CCX733 and CCX771 were indistinguishable from the control compound CCX704. (TIF) [file pone.0033005.s007.tif]
